# Enhanced Photovoltaic Performance of Poly(3,4-Ethylenedioxythiophene)Poly(*N*-Alkylcarbazole) Copolymer-Based Counter Electrode in Dye-Sensitized Solar Cells

**DOI:** 10.3390/polym16202941

**Published:** 2024-10-20

**Authors:** Sherif Dei Bukari, Aliya Yelshibay, Bakhytzhan Baptayev, Mannix P. Balanay

**Affiliations:** 1 Chemistry Department, Nazarbayev University, 53 Kabanbay Batyr Ave., Astana 01000, Kazakhstan; sherif.bukari@nu.edu.kz (S.D.B.); aliya.yelshibay@nu.edu.kz (A.Y.); 2 National Laboratory Astana, Nazarbayev University, 53 Kabanbay Batyr Ave., Astana 010000, Kazakhstan

**Keywords:** 3,4-ethylenedioxythiophene, carbazole, copolymers, electropolymerization, Pt-free counter electrodes

## Abstract

Conducting polymers are emerging as promising alternatives to rare and expensive platinum for counter electrodes in dye-sensitized solar cells; due to their ease of synthesis, they can be chemically tuned and are suitable for roll-to-roll production. Among these, poly (3,4-ethylenedioxythiophene) (PEDOT)-based counter electrodes have shown leading photovoltaic performance. However, certain conductivity issues remain that affect the effectiveness of these counter electrodes. In this study, we present an electropolymerized PEDOT and poly(*N*-alkylated-carbazole) copolymer as an efficient electrocatalyst for the reduction in $I_{3}^{-}$ in dye-sensitized solar cells. Copolymerization with *N*-alkylated carbazoles significantly increases the conductivity of the polymer film and facilitates rapid charge transport at the interface between the polymer electrode and the electrolyte. The length of the alkyl substituents also plays a crucial role in this improvement. Electrochemical analysis showed a reduction in charge transport resistance from 3.31 Ω·cm^2^ for PEDOT to 2.26 Ω·cm^2^ for the PEDOT:poly(*N*-octylcarbazole) copolymer, which is almost half the resistance of a platinum-based counter electrode (4.12 Ω·cm^2^). Photovoltaic measurements showed that the solar cell with the PEDOT:poly(*N*-octylcarbazole) counter electrode achieved an efficiency of 8.88%, outperforming both PEDOT (7.90%) and platinum-based devices (7.57%).

## 1. Introduction

The urgent need to replace fossil fuels as a primary energy source and concerns about resource depletion, pollution and negative effects on the climate have led the scientific community to intensify research into alternative green and renewable energy sources. Among these alternatives, photovoltaic technology has shown particular promise. Over the past three decades, dye-sensitized solar cells (DSSCs) have attracted much attention in the field of photovoltaics, mainly because of their relatively high efficiency, low cost, excellent low-light performance, ease of fabrication and design flexibility [1,2,3]. DSSCs, a third-generation photovoltaic technology, are photoelectrochemical cells consisting of an anode, a redox electrolyte and a counter electrode (CE). The anode consists of a thin film of nano-sized, mesoporous, semiconducting TiO_2_ coated with a monolayer of light-absorbing dye. The counter electrode is a thin film of an electrocatalytic material, such as platinum. These two electrodes are arranged parallel to each other, with the space between them filled with a redox electrolyte. When photons are absorbed, the dye is photoexcited and injects its electrons into the TiO_2_ conduction band, which then diffuses to the back contact. The oxidized dyes are regenerated by the redox electrolyte. The counter electrode is a crucial component of dye solar cells. It is responsible for collecting electrons from the external circuit and facilitating the reduction in the redox couple in the electrolyte. This function has a significant impact on the cost, long-term stability and overall efficiency of the solar cell [4]. Traditionally, platinum has been used as a CE material, but its rarity and high cost have hindered the commercialization of DSSCs. Consequently, the search for alternative CE materials that are cost-effective and have properties comparable to or even superior to those of Pt has become a major challenge for the scientific community and is essential for the advancement of DSSC technology. Various potential substitutes for Pt have been investigated, including carbonaceous materials, conducting polymers (CPs), metal compounds and composites [3,5]. Among these, conducting polymers have emerged as promising candidates due to their high conductivity, good stability, low cost and effective catalytic activity for the reduction in redox couples [6]. In particular, poly(3,4-ethylenedioxythiophene) (PEDOT) has attracted great interest due to its excellent stability, electrical conductivity, catalytic performance and ease of preparation [7]. PEDOT counter electrodes are usually prepared via electropolymerization [8] or vapor phase polymerization [9], methods that allow additives to be incorporated into the polymer matrix to produce doped PEDOT films. Early research on Pt-free PEDOT-based counter electrodes, such as the work of Yanagida et al. [10], has emphasized the importance of the type of dopant for the development of effective PEDOT-based CEs for iodide/triiodide (I^−^/I_3_^−^) DSSCs. Various strategies have been employed to improve the performance of PEDOT CEs, including optimization of preparation methods [9], modification of solvents [11] and development of composites [12,13]. However, copolymerization of PEDOT is less frequently reported, providing a potential avenue for further research.

Carbazole polymers have been intensively researched in recent decades due to their unique properties as conducting polymers, which are primarily due to their higher redox potential [14]. Due to their exceptional light absorption and efficient hole transport, they are ideally suited for various applications, such as light-emitting diodes [15], transistors [16], sensors [17] and photovoltaics [18]. These advantageous properties have led us to investigate the copolymerization of carbazoles with 3,4-ethylenedioxythiophene for use as counter electrode materials in dye-sensitized solar cells.

In this study, we investigate the potential of copolymers of 3,4-ethylenedioxythiophene and three different *N*-alkylcarbazole derivatives to improve the performance of counter electrodes. Our copolymers achieve impressive photon conversion efficiencies (PCEs) of up to 8.88%, outperforming both PEDOT-based (7.90% PCE) and platinum-based (7.57% PCE) counter electrodes. These results highlight the significant efficiency gains that can be achieved through copolymerization. Furthermore, this approach represents a promising way to optimize energy conversion devices by refining the electronic properties and interfacial interactions of the active materials.

## 2. Materials and Methods

Materials and reagents: Chemical reagents, fluorine-doped tin oxide (FTO) coated glass slides (2.2 mm thick, surface resistivity approximately 7 Ω/sq), titanium (IV) isopropoxide, TiO_2_ reflector paste (Greatcell Solar WER2-O, 150–250 nm), N719 dye (CAS Number: 207347-46-4), hexachloroplatinic-acid-based Pt paste (Greatcell Solar PT1) were purchased from Sigma-Aldrich (Queanbeyan, Australia). The methoxypropionitrile (MPN)-based iodide/triiodide redox electrolyte (DN-OD03) and transparent TiO_2_ paste (DN-EP03, 18–20 nm) were acquired from Dyenamo (Stockholm, Sweden). 

Synthesis of 9-butyl-9H-carbazole (CbzC4): Carbazole (24 mmol, 4.0 g), butyl bromide (39.98 mmol, 4.31 mL) and 8.0 g of NaOH were mixed together in a flask containing 60 mL of dimethyl sulfoxide (DMSO). The resulting mixture was stirred at room temperature. Upon completion of the reaction (monitored by TLC), the mixture was neutralized with 10% diluted HCl. The combined organic extract was washed several times with distilled water before separation. Following the chloroform extraction, the organic phase was evaporated using a rotary evaporator. Purification through column chromatography using an n-hexane/ethyl acetate (9:1) eluent system afforded the pure product as crystals (4.7 g, 88% yield), which were subsequently dried under reduced pressure. ^1^H NMR (500 MHz, acetone-*d*_6_, ppm) δ 8.11 (d, J = 7.7 Hz, 2H), 7.53 (d, J = 8.3 Hz, 2H), 7.42 (t, J = 7.7 Hz, 2H), 7.17 (t, J = 7.5 Hz, 2H), 4.40 (t, J = 7.2 Hz, 2H), 1.82 (p, J = 7.6 Hz, 2H), 1.37 (dq, J = 14.8, 7.4 Hz, 2H), 0.90 (t, J= 7.4 Hz, 3H).

Synthesis of 9-hexyl-9H-carbazole (CbzC6): The synthesis of CbzC6 was conducted with some modifications based on a previously described procedure [19]. Initially, 8.0 g of NaOH was dissolved in 60 mL of DMSO in a dried 250 mL round-bottom flask, followed by stirring for 30 min. To this mixture, 4.0 g of carbazole (24 mmol) was added, and then, 5.6 mL of hexylbromide (39.98 mmol) was slowly added dropwise. Afterward, cold de-ionized water was introduced to precipitate the product, which was then separated via filtration, washed three times with DI water and then dried. The purified product was obtained as crystals through column chromatography using an n-hexane/ethyl acetate (9:1) eluent system and subsequently dried under reduced pressure, yielding 5.7 g of the pure product (94% yield). ^1^H NMR (500 MHz, acetone-d6, ppm) δ 8.11 (d, J = 7.8 Hz, 2H), 7.49 (d, J = 8.3 Hz, 2H), 7.45–7.37 (m, 2H), 7.18 (t, J = 7.5 Hz, 2H), 4.32 (t, J = 7.3 Hz, 2H), 1.79 (p, J = 7.5 Hz, 2H), 1.44–1.01 (m, 6H), 0.80 (t, J = 7.2 Hz, 3H).

Synthesis of 9-octyl-9H-carbazole (CbzC8): In a manner similar to the synthesis of CbzC4, a mixture of carbazole (1.67 g), bromooctane (2.88 g) and NaOH (0.1 mol, 4.00 g) was prepared in a flask containing 30 mL of DMSO. The mixture was stirred at room temperature for approximately 3 h. Upon completion of the reaction, monitored by TLC, the mixture was neutralized with 10% diluted HCl, and the product was extracted with chloroform (15 mL, 5 times). The combined organic extracts were washed multiple times with distilled water and separated. The organic extract was first treated with anhydrous sodium sulfate and stirred at room temperature to eliminate any residual water. After filtration, the organic phase underwent evaporation using a rotary evaporator. The dried extract was then filtered, purified via column chromatography using an n-hexane/ethyl acetate solvent ratio of 10:1, and the eluent was evaporated under vacuum to yield 2.32 g (83% yield) of the compound as a yellow oil. ^1^H NMR (500 MHz, acetone-d6) δ 8.11 (d, J = 7.8 Hz, 2H, ppm), 7.54 (d, J = 8.2 Hz, 2H), 7.47–7.37 (m, 2H), 7.17 (t, J = 7.4 Hz, 2H), 4.40 (t, J = 7.2 Hz, 2H), 1.85 (p, J = 7.5 Hz, 2H), 1.47–1.07 (m, 10H), 0.81 (t, 3H).

Fabrication of counter electrodes: The procedure began with FTO substrates, each measuring 1.5 cm by 2.0 cm, which were initially sonicated in piranha solution (H_2_SO_4_: H_2_O_2_ with 3:1 ratio) and cleaned thoroughly in soapy water for 30 min. Subsequently, they were rinsed with deionized water and subjected to a 30 min sonication in ethanol before being dried in an oven at 70 °C for at least 2 h. The copolymerization process followed modifications based on the methods described by Cansu-Ergun and colleagues [20]. For the copolymerization, 3,4-ethylenedioxythiophene (EDOT), CbzC4, CbzC6 and CbzC8 were separately dissolved in DCM to prepare 10 mM solutions of each monomer. These solutions were then combined in separate vials at a ratio of 9.5:0.5 (EDOT:CbzC4, EDOT:CbzC6 and EDOT:CbzC8; v:v). The resulting solutions of carbazole derivatives and EDOT were electrodeposited onto the pre-cleaned FTO substrates (1.5 cm × 2 cm) using a DCM-tetrabutylammonium hexafluorophosphate electrolytic medium. Additionally, a PEDOT-based counter electrode was produced under identical conditions for comparison. Furthermore, three Pt counter electrodes were fabricated by applying Pt paste onto pre-cleaned and dried FTO substrates, followed by sintering at 500 °C for 30 min, to serve as control devices.

Fabrication of photoanode and dye-sensitized solar cell: The FTO slides were first cleaned and dried according to previous procedures. They were then immersed in a 50 mM solution of titanium (IV) isopropoxide in HCl for 30 min, followed by sintering at 500 °C for 30 min, to create a compact TiO_2_ coating. Next, a transparent TiO_2_ paste was applied over the compact layer and air-dried for 1 h. The film underwent stepwise sintering at temperatures of 125 °C, 325 °C, 425 °C and finally 500 °C. Following this, a scattering layer of TiO_2_ paste was applied on top of the transparent TiO_2_ and air-dried for 1 h. The samples were then sintered again at 125 °C, 325 °C, 425 °C and 500 °C. After cooling to temperatures between 70 °C and 80 °C, the electrodes were immersed in a 0.25 mM N719 dye solution composed of acetonitrile and tert-butanol (1:1) solvent and left to soak for 24 h. To prepare the photoanodes, excess dye molecules were removed from the TiO_2_ electrodes through washing with ethanol. Subsequently, the electrolyte was drop-cast onto the dye-loaded TiO_2_ electrode, and a counter electrode was placed on top, separated by a double-sided tape acting as a spacer to prevent short-circuiting.

Characterization and measurement: ^1^H NMR analysis was conducted using a JNM-ECA 500 MHz spectrometer (JEOL Ltd., Tokyo, Japan). The morphologies of the PEDOT and copolymer counter electrodes were examined with a JEOL JSM-IT200 (LA) scanning electron microscope (Tokyo, Japan). To assess the electrochemical properties of the counter electrodes (CEs), a PalmSens4 Potentiostat/Galvanostat (PalmSens Technologies BV, Houten, Utrecht, The Netherlands) was utilized. The catalytic activity of the synthesized CEs was evaluated through cyclic voltammetry (CV) and electrochemical impedance spectroscopy (EIS). The EIS measurements were performed at a constant temperature of 20 °C with an AC signal amplitude of 20 mV, covering a frequency range from 0.1 to 105 Hz under 0 V DC bias in the dark, simulating open-circuit conditions in ambient atmosphere. For the CV analysis, a three-electrode system was employed using an acetonitrile electrolyte solution that contained 10 mM LiI, 1 mM I_2_ and 0.1 M LiClO_4_. In this setup, a platinum wire served as the counter electrode; a silver–silver chloride (Ag/AgCl) reference electrode (in 3 M NaCl solution) was used; and the as-fabricated CE acted as the working electrode. Photovoltaic measurements were conducted using the Dyenamo Toolbox (DN-AE01) from Dyenamo AB (Stockholm, Sweden).

## 3. Results and Discussion

### 3.1. Synthesis of N-Alkylcarbazole Monomers

Three monomers—9-butyl-9*H*-carbazole (CbzC4), 9-hexyl-9*H*-carbazole (CbzC6) and 9-octyl-9*H*-carbazole (CbzC8)—were synthesized from the simple precursor 9*H*-carbazole (Cbz) according to a modified method described in the literature [19]. The synthesis of CbzC4 and CbzC6 resulted in solid products with an overall yield of 88% and 94%, respectively. In contrast, the synthesis of CbzC8 rendered a liquid product (yellow oil) with a yield of 83% after drying under reduced pressure. The structures of all three monomers were confirmed via ^1^H NMR analysis. Figure S1 in the Supporting Information displays the ^1^H NMR spectrum of 9*H* carbazole. Additionally, Figures S2–S4 present the corresponding spectra of the synthesized monomers CbzC4, CbzC6 and CbzC8, respectively. In particular, the disappearance of the N–H peak at a chemical shift of 10.34 ppm (Figure S1) and the presence of C–H alkyl shifts between 0.8 ppm and 5.0 ppm (Figures S2–S4) indicate successful deprotonation of the nitrogen atom in the Cbz structure and alkylation at the N-position of the carbazole substrate.

### 3.2. Characterization of Copolymer-Based Counter Electrodes

#### 3.2.1. Raman Characterization

**Figure 1:**
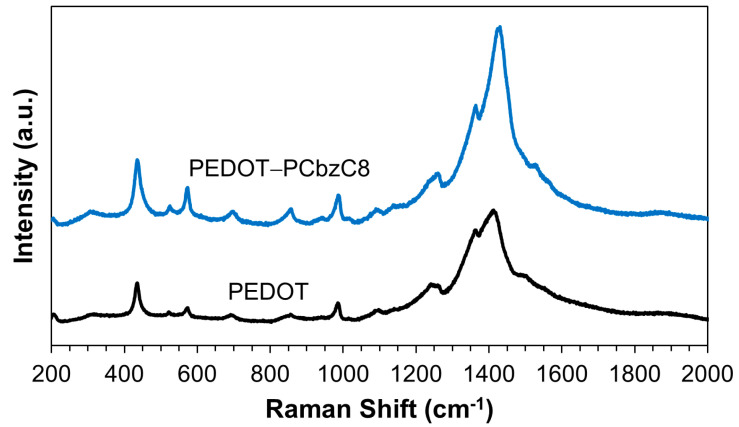
Raman spectra of the PEDOT-PCbzC8 and PEDOT films.

Figure 1 illustrates the Raman spectra for both pristine and doped PEDOT films. The spectra reveal that the peak for pristine PEDOT is located at 1430.2 cm^−1^, while the doped PEDOT shows a peak at 1410.1 cm^−1^. These peaks correspond to the symmetric stretching of the benzoid Cα = Cβ bonds. The observed red shift in the peak position for the doped PEDOT suggests enhanced crystallinity resulting from the doping process. This red shift is consistent across all observed peaks, indicating successful doping. Specifically, the peaks at 1363.9 cm^−1^ for doped PEDOT and 1361.9 cm^−1^ for pristine PEDOT are attributed to the Cβ–Cβ stretching deformations. The Cα–Cα inter-ring stretching vibrations are observed at 1260.1 cm^−1^ for doped PEDOT and 1259.4 cm^−1^ for pristine PEDOT. Additionally, the oxyethylene ring deformation vibrations appear at 987.4, 856.9 and 573.2 cm^−1^ for doped PEDOT and at 985.3, 855.6 and 572.4 cm^−1^ for pristine PEDOT. The signals at 695.6 cm^−1^ for doped PEDOT and 691.1 cm^−1^ for pristine PEDOT correspond to the symmetric C–S–C deformations [21]. 

#### 3.2.2. Morphology of Polymer-Based Counter Electrodes

**Figure 2:**
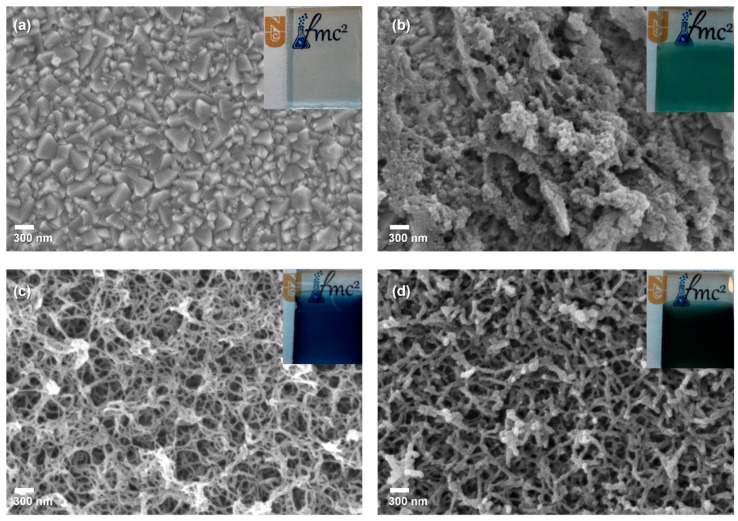
SEM images of (**a**) bare-FTO, (**b**) PCbzC8, (**c**) PEDOT and (**d**) PEDOT-PCbzC8 counter electrodes. The inset features a photograph of the actual counter electrode.

FE-SEM analysis was utilized to examine the surface structures of the counter electrode films PCbzC8, PEDOT and the doped polymer PEDOT-PCbzC8, as depicted in Figure 2. Figure 2a illustrates the surface structure of an uncoated transparent conducting oxide substrate, specifically fluorine-doped tin oxide, after being cut. The inset in Figure 2a highlights a precisely cut FTO glass with smooth edges, designed to minimize sheet resistance and reduce overall series resistance. In Figure 2b, the poly(9-octyl-9*H*-carbazole) (PCbzC8) film appears non-homogeneous with a rough surface compared to the other polymer films, featuring a compact and relatively low-porous structure. This increased surface roughness is anticipated to enhance electrocatalytic activity for the I^−^/I_3_^−^ redox pair and decrease the charge transfer resistance at the CE/electrolyte interface [22,23], thereby potentially improving both conductivity and electrocatalytic performance of the copolymer counter electrode. Figure 2c shows the PEDOT film with a highly porous, mesh-like structure, indicating a large effective surface area that could enhance electrocatalytic activity [24]. However, the presence of large pores suggests a lack of well-oriented charge transfer channels within the layer [24,25]. To address this issue, the PEDOT-PCbzC8 doped polymer film, depicted in Figure 2d, combines the structural benefits of both materials. The copolymerization of EDOT with the CbzC8 monomer resulted in a nanotube-like structure with more oriented charge transfer channels, expected to enhance the performance of the copolymer film. Detailed cross-sectional images of PEDOT, PCbzC8 and PEDOT-PCbzC8 are presented in Figure S5 of the Supporting Information.

The insets in Figure 2 show images of the thin films on FTO. The PCbzC8 film has a blue-green (turquoise) color (Figure 2b), while the PEDOT film appears dark blue (Figure 2c). After copolymerization, the color of the PEDOT-PCbzC8 film changed to a dark blue-green (Figure 2d). This color change prompted us to investigate the light absorption properties of the films. The resulting UV–Vis absorption spectra and the corresponding Tauc plots are shown in Figure S6. Analysis of the Tauc plots revealed that the polymer band gap (E_g_) for PCbzC8 is 2.2 eV. A band gap of 3.4 eV was calculated for PEDOT, while the copolymer band gap of 3.2 eV lies between those of PEDOT and PCbzC8.

### 3.3. Photovoltaic Performance and Stability

**Figure 3:**
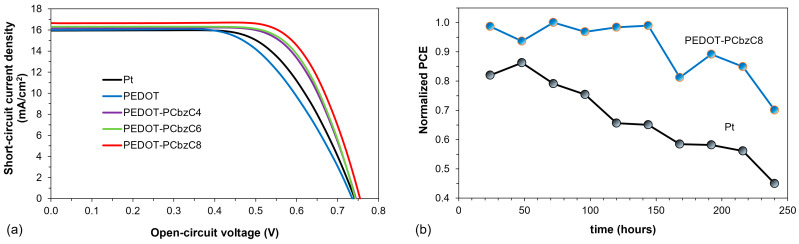
(**a**) J–V curve of dye-sensitized solar cells and (**b**) stability of the power conversion efficiencies of dye-sensitized solar cells with Pt and copolymer counter electrodes.

Table 1 summarizes the photovoltaic characteristics of various samples based on standard deviation data from three dye-sensitized solar cell devices tested under white light LED illumination at 100 mW/cm^2^. We first evaluated the performance of DSSCs featuring different PEDOT-carbazole derivative copolymer films—specifically PEDOT-PCbzC4, PEDOT-PCbzC6 and PEDOT-PCbzC8—compared to bare PEDOT and platinum counter electrodes. The photocurrent density–voltage (J–V) curves for all five counter electrodes are illustrated in Figure 3a. Both Figure 3a and Table 1 indicate a consistent increase in the short-circuit current density (J_SC_) and fill factor (FF) for the copolymeric CEs. This improvement may be attributed to the lengthening of the alkyl chain attached to the carbazole nitrogen atom, which reduces the solubility of the carbazole monomer in acetonitrile. This decreased solubility subsequently affects the copolymer film’s solubility in the acetonitrile-based iodide electrolyte. Consequently, the mechanical strength of copolymer films with longer *N*-alkyl chains is likely enhanced, resulting in better contact with the FTO substrate. This is corroborated by the observation of the lowest ohmic series resistance (*R_S_*) in the DSSC featuring the PEDOT-PCbzC8 counter electrode [24]. The PEDOT-PCbzC8 copolymer CE demonstrates the highest short-circuit current density of 16.65 ± 0.27 mA/cm^2^, a fill factor of 0.71 ± 0.07 and a power conversion efficiency of 8.88 ± 0.09%, surpassing the performance of other copolymer CEs, as well as the PEDOT and Pt CEs. Figure 3a also presents the J–V curves for DSSCs with various CEs, including Pt, PEDOT and the highest-performing PEDOT-PCbzC8, all under 100 mW/cm^2^ illumination. Notably, the DSSCs with polymer-based electrodes (PEDOT ~7.90 ± 0.04% and PEDOT-PCbzC8 ~8.88 ± 0.09%) demonstrate superior photoelectric performance compared to those using a Pt electrode (~7.57 ± 0.20%). Several factors contribute to the enhanced photoelectric performance of DSSCs with polymer counter electrodes. The morphology of the CE is critical, as it significantly influences the electrocatalyst’s ability to reduce the redox mediator. The porous structure of PEDOT and PEDOT-PCbzC8 films increases the active surface area of the electrodes while improving stability by trapping the liquid electrolyte within the micropores. A larger surface area for the counter electrode leads to a significant enhancement in the iodine/triiodide redox reaction at the electrode interface, thus boosting the photovoltaic performance of the DSSCs [24].

The stability of the power conversion efficiencies of dye-sensitized solar cells with Pt and PEDOT-PCbzC8 counter electrodes was evaluated over 200 h under ambient conditions. The results, illustrated in Figure 3b, show that the solar cells with PEDOT-PCbzC8 counter electrodes retained more than 70% of their initial PCE after 200 h of testing. In contrast, the PCE of DSSCs with Pt counter electrodes decreased by nearly 50% over the same period. This study highlights that PEDOT-PCbzC8 counter electrodes exhibit greater stability compared to traditional Pt counter electrodes. 

### 3.4. Electrochemical Analysis of Polymer-Based Counter Electrodes

#### 3.4.1. Electrochemical Impedance Spectroscopy and Tafel Polarization Analysis

**Figure 4:**
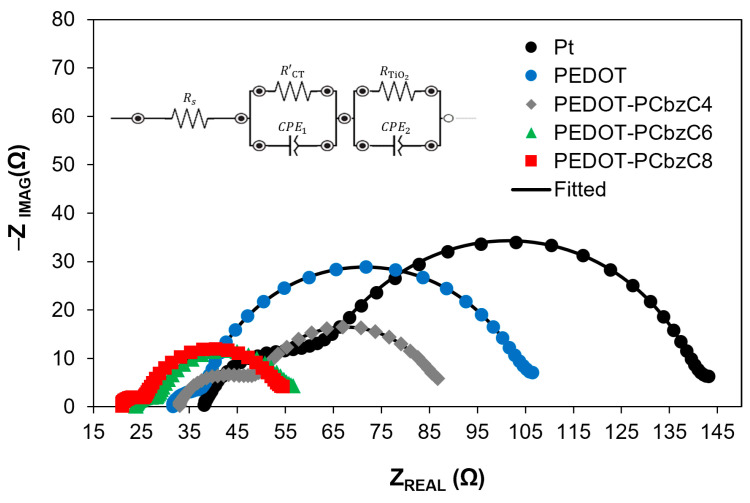
Nyquist plots (inset is the equivalent electrical circuit used for fitting) of dye-sensitized solar cells with Pt, PEDOT and copolymer counter electrodes.

To investigate the catalytic performance of dye-sensitized solar cells with various counter electrodes, we employed electrochemical impedance spectroscopy, as illustrated in Figure 4. The impedance spectra were analyzed using an electrical equivalent circuit, which is detailed in the inset of Figure 4. The series resistance (*R_S_*), the charge transfer resistance at the CE/electrolyte interface (*R’_CT_*) and the resistance at the TiO_2_/dye/electrolyte interface ($R_{TiO_{2}}$) are represented by the X-axis intercept of the curve, the smaller semicircle at higher frequencies and the larger semicircle, respectively, across all three curves. A lower charge transfer resistance indicates superior electrocatalytic activity of the counter electrode in reducing triiodide species. Among the polymer-based counter electrodes, those with PEDOT-PCbzC8 exhibit a smaller charge transfer resistance at the CE/electrolyte interface. Specifically, the *R′_CT_* value decreases from 18.21 Ω for the Pt device to 14.00 Ω for the PEDOT-PCbzC8 device, as shown in Table 1. The reduced resistance at the interface between the electrode and the electrolyte facilitates electron transport and increases the photocurrent density, which can accelerate the regeneration process of the dye, as the iodine ions can accumulate closer to the dye. Consequently, this effect contributes to the higher current density observed in copolymeric CEs. The EIS analysis shows that the PEDOT-PCbzC8 CE has a higher catalytic activity than other copolymer CEs, such as PEDOT-PCbzC4, PEDOT-PCbzC6 and PEDOT, all of which perform better than the Pt CE. 

**Figure 5:**
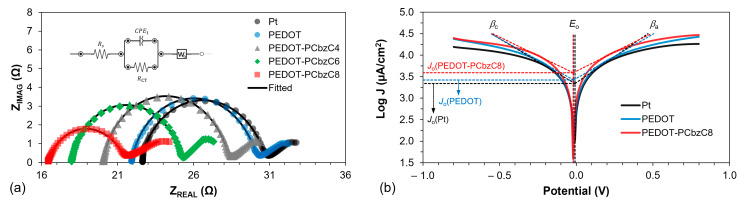
(**a**) Nyquist plot of symmetrical dummy cells (inset is the equivalent electrical circuit used for fitting the Nyquist plots); (**b**) Tafel polarization curves of Pt, PEDOT and PEDOT-PCbzC8.

The electron transfer processes at the counter electrode/electrolyte interface for Pt-, PEDOT- and copolymer-based DSSCs were further investigated using the Nyquist plots of symmetrical dummy cells, as shown in Figure 5a, which presents the Nyquist plots for all five counter electrodes, with its inset illustrating the equivalent circuit used to fit the electrochemical impedance spectra. In this circuit, *Rs* represents the ohmic series resistance between the substrate (FTO) and the catalytic film. The onset of the first semicircle in the high-frequency region can be used to determine the *R_S_* value. A lower *R_S_* indicates better contact of the catalytic film with the FTO substrate, which enhances film conductivity and results in a higher fill factor for the DSC device. As shown in Table 1, the PEDOT-PCbzC8 copolymer electrode exhibits a lower *Rs* value compared to DSSCs with only PEDOT or Pt, leading to a higher FF value, consistent with its *Rs* value. Additionally, the radius of the first semicircle in the middle-frequency zone provides an estimate of the charge transfer resistance (*R_CT_*) at the electrode/electrolyte interface. A lower *R_CT_* value indicates better electrocatalytic activity, contributing to a higher J_SC_ of the DSSC. PEDOT-PCbzC8 demonstrated the lowest *R_CT_* value of 2.26 Ω·cm^2^, whereas PEDOT and Pt had *R_CT_* values of 3.31 Ω·cm^2^ and 4.12 Ω·cm^2^, respectively. 

Tafel measurements are valuable for assessing electrocatalyst performance. The Tafel polarization curve (Figure 5b), which typically comprises three regions—polarization, Tafel and diffusion—is particularly useful for evaluating the counter electrode’s effectiveness. Among these, the Tafel and diffusion regions offer insights into crucial parameters, such as the exchange current density (*J_o_*). Notably, there is an inverse relationship between *J_o_* and the charge transfer resistance at the counter electrode/electrolyte interface, as described by Equation (1).

(1) $$J_{o}=\frac{RT}{nFR_{CT}}$$

where *R* is the universal gas constant; *T* is the absolute temperature; *n* is the number of electrons; and *F* is the Faraday constant. The inverse relationship depicted in the equation indicates that a higher exchange current density results in a lower charge transfer resistance, which in turn improves electrocatalytic activity.

Figure 5b illustrates that the Tafel plot for PEDOT-PCbzC8 exhibits a steeper slope compared to PEDOT and Pt. Specifically, the cathodic slope (β_c_) for PEDOT-PCbzC8 is 577 mV/decade, and the anodic slope (β_a_) is 560 mV/decade. In comparison, PEDOT has a β_c_ of 500 mV/decade and a β_a_ of 486 mV/decade, while Pt shows a β_c_ of 471 mV/decade and a β_a_ of 410 mV/decade. This larger slope indicates that PEDOT-PCbzC8 has a higher exchange current density, with J_O_ for PEDOT-PCbzC8 at approximately 3.91 mA/cm^2^ compared to 2.65 mA/cm^2^ for PEDOT and 2.18 mA/cm^2^ for Pt. This is consistent with the observed *R_CT_* of the electrodes presented in Table 1. This observation confirms that PEDOT-PCbzC8 exhibits superior electrocatalytic activity compared to both PEDOT and Pt counter electrodes.

#### 3.4.2. Cyclic Voltammetry Analysis

**Figure 6:**
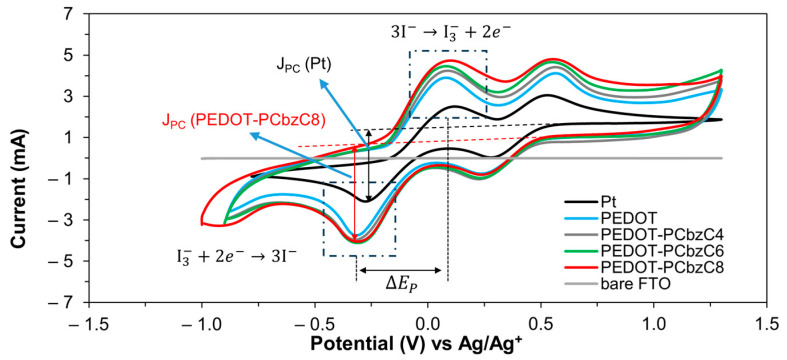
Cyclic voltammograms of all counter electrodes.

Cyclic voltammetry was utilized to evaluate the electrocatalytic performance of the counter electrodes for triiodide ion reduction. Figure 6 illustrates the cyclic voltammograms from a three-electrode electrochemical cell, where Pt, PEDOT, PEDOT-PCbzC8 and two other copolymer CEs were employed as working electrodes. The measurements were conducted in an acetonitrile solution containing LiClO_4_ as the supporting electrolyte, along with LiI and I_2_, at a scan rate of 100 mV/s. At the working electrode, iodide ion oxidation occurs, as depicted in Equation (2), while at the counter electrode, triiodide ion reduction takes place, as outlined in Equation (3) [25].

(2) $$3I^{-}\to I_{3}^{-}+2e^{-}$$

(3) $$I_{3}^{-}+2e^{-}\to 3I^{-}$$

The electrocatalytic performance of counter electrodes is assessed using two key parameters: peak separation (ΔE_P_) and cathodic peak current density (J_PC_). ΔE_P_ measures the kinetic redox capability of a CE for the I^−^/I_3_^−^ couple, where a lower ΔE_P_ suggests better performance [25]. Conversely, a higher J_PC_ value indicates stronger electrocatalytic ability. As shown in Figure 6, the polymer CEs exhibit a slightly larger ΔE_P_ compared to Pt CEs, which implies a marginally higher overpotential for the catalytic processes of PEDOT and PEDOT-PCbzC8 [26]. Despite this, the significantly higher J_PC_ values of the polymer CEs overshadow the differences in ΔE_P_. As a result, the DSSC device equipped with a PEDOT-PCbzC8 CE achieves a short-circuit current density of 16.65 mA/cm^2^, surpassing the J_SC_ values of PEDOT (16.02 mA/cm^2^) and Pt (15.95 mA/cm^2^) CEs. This outcome aligns with the observation that PEDOT-PCbzC8 CE exhibits the highest J_PC_ value among the tested CEs. 

**Figure 7:**
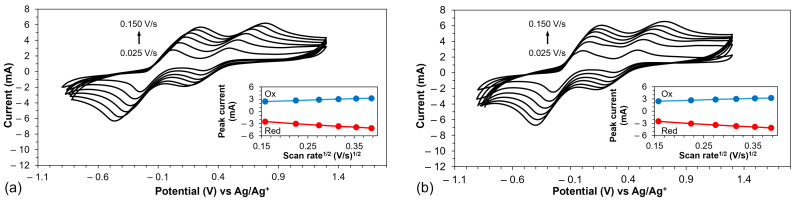
Cyclic voltammogram of (**a**) PEDOT and (**b**) PEDOT-PCbzC8 CE at different scan rates. The insets show the scan rate dependence of the peak current of the electrodes.

Figure 7 displays the cyclic voltammograms of PEDOT and PEDOT-PCbzC8 across various scan rates. For additional details, Figure S7 in the Supporting Information presents the CVs of PEDOT-PCbzC4 and PEDOT-PCbzC6 under similar conditions. These figures illustrate that as the scan rate increases, the intensity of the redox peak signals also rises. Furthermore, the insets in Figure 7 and Figure S7 show the relationship between the peak current and the scan rate for the polymer counter electrodes. The results reveal a linear relationship between the square root of the scan rate and the redox peak current, indicating that ion diffusion is a significant factor in triiodide reduction and suggesting that there is no chemical reaction between the redox electrolyte and the electrocatalyst.

In summary, the electrocatalytic properties of the counter electrodes, as assessed through cyclic voltammetry, Tafel polarization plots and electrochemical impedance spectroscopy, align well with the photovoltaic parameters of their respective dye-sensitized solar cells. The newly developed PEDOT-PCbzC8 copolymer-based electrocatalyst demonstrated exceptional efficiency in catalyzing the reduction of triiodide to iodide in dye-sensitized solar cells. Its superior electrocatalytic activity allows it to outperform traditional platinum-based counter electrodes in DSSCs. Compared to other polymer and copolymer composite-based counter electrodes for DSSCs, as shown in Table 2, the PEDOT-PCbzC8 copolymer-based electrocatalyst exhibits the lowest charge transfer resistance and ranks among the highest in terms of power conversion efficiency.

## 4. Conclusions

A straightforward anodic electrodeposition method was used to successfully create the PEDOT-PCbzC8 copolymer, which was then utilized as a counter electrode in a dye-sensitized solar cell. Scanning electron microscopy analysis revealed that the PEDOT-PCbzC8 copolymer features a nanotube-like surface morphology that combines the rough texture of PCbzC8 with the highly porous, mesh-like surface of PEDOT. Among the three copolymer counter electrodes tested in DSSCs, the device with the PEDOT-PCbzC8 counter electrode exhibited the highest power conversion efficiency of 8.88 ± 0.09%. By comparison, the devices with PEDOT-PCbzC6 and PEDOT-PCbzC4 counter electrodes achieved PCE values of 8.52 ± 0.04% and 8.00 ± 0.06%, respectively. This trend indicates that increasing the length of the *N*-alkyl chain in the copolymer monomers enhances power conversion efficiency. The increased bulkiness of the copolymer likely reduces its solubility in the acetonitrile solvent of the iodide-based electrolyte. Furthermore, DSSCs using these copolymer counter electrodes demonstrated higher PCE values compared to those with PEDOT (7.90 ± 0.04%) and platinum (7.57 ± 0.2%) counter electrodes. The superior electrocatalytic properties of the copolymers are supported by the cathodic peak current densities observed in cyclic voltammograms and the exchange current densities measured in Tafel polarization plots. Therefore, PEDOT-PCbzC8 is a promising electrocatalytic material for DSSCs and offers a cost-effective alternative to platinum.

## Figures and Tables

**Table 1:** Photovoltaic and electrochemical performance parameters of Pt- and polymer-based DSSCs under white LED illumination at an intensity of 100 mW/cm^2^.

CE	η (%)	V_OC_ (V)	J_SC_ (mA/cm^−2^)	FF	$R_{CT}$ (Ω·cm^2^)	$R_{S}$ (Ω)	${R^{\prime }}_{CT}$ (Ω)	$R_{TiO_{2}}$ (Ω)
Pt	7.57 ± 0.20	0.74 ± 0.02	15.95 ± 0.4	0.64 ±0.03	4.12	31.00	18.21	74.99
PEDOT	7.90 ± 0.04	0.74 ± 0.01	16.02 ± 0.16	0.68 ± 0.01	3.31	29.41	17.89	59.93
PEDOT-CbzC4	8.00 ± 0.06	0.72 ± 0.01	16.24 ± 0.15	0.69 ± 0.01	3.06	26.38	17.55	40.54
PEDOT-CbzC6	8.52 ± 0.04	0.74 ± 0.01	16.31 ± 0.03	0.70 ± 0.003	2.50	24.00	16.71	43.23
PEDOT-CbzC8	8.88 ± 0.09	0.76 ± 0.01	16.65 ± 0.27	0.71 ± 0.07	2.26	21.00	14.00	46.79

**Table 2:** Comparison of photovoltaic performance of dye-sensitized solar cells utilizing various polymer composites as counter electrodes, with I^−^/I_3_^−^ electrolyte and N719 dye, under illumination of 100 mW/cm^2^.

Counter Electrodes	Method of Application	R_CT_	PCE (%)	Reference
Polyvinyl alcohol nanofibers coated with PEDOT	Electropolymerization	–	2.11	[27]
PolyPyrrole-SrTiO_3_	Oxidative polymerization	46.4	2.52	[28]
Bottom ash/PEDOT:PSS/polyvinylpyrrolidone	Doctor blade	276.70	2.70	[29]
Polyoxometalate-doped PEDOT	Electropolymerization	7.67	5.81	[30]
Polyaniline-graphene oxide	In situ polymerization	–	6.12	[31]
Reduced graphene oxide/Polypyrrole/PEDOT composite	Polymerization and electrodeposition	–	7.10	[32]
Cu-polypyrrole-MWCNT	Electrochemical synthesis	4.31	7.10	[33]
Polyaniline-graphene	In situ polymerization	20.1	7.45	[31]
Poly(spiroBiProDOT)	Electrochemical polymerization	17.6	7.90	[34]
PEDOT-poly(*N*-butylcarbazole)	Electropolymerization	3.06	8.00	This work
NiS-PEDOT-PSS	Simple mixing	0.46	8.18	[35]
TiO_2_-PEDOT-PSS	Simple mixing	1.3	8.49	[36]
PEDOT-poly(*N*-hexylcarbazole)	Electropolymerization	2.50	8.52	This work
PEDOT-poly(*N*-octylcarbazole)	Electropolymerization	2.26	8.88	This work

## Data Availability

The original contributions presented in the study are included in the article/Supplementary Material. Further inquiries can be directed to the corresponding authors.

## Supplementary Materials

The following supporting information can be downloaded at: https://www.mdpi.com/article/10.3390/polym16202941/s1, Figure S1: ^1^H NMR spectrum of 9*H*-carbazole in acetone-*d*_6_; Figure S2: ^1^H NMR spectrum of CbzC4 in acetone-*d*_6_; Figure S3: ^1^H NMR spectrum of CbzC6 in acetone-*d*_6_; Figure S4: ^1^H NMR spectrum of CbzC8 in acetone-*d*_6_; Figure S5: Cyclic voltammogram of (a) PEDOT-PCbzC4 and (b) PEDOT-PCbzC6 CE at different scan rates. The insets show the scan rate dependence of the peak current of the electrodes; Figure S6: (a) UV–Vis absorption and (b) Tauc plot of polymer thin films on FTO glass substrate; Figure S7: Cyclic voltammogram of (a) PEDOT-PCbzC4 and (b) PEDOT-PCbzC6 CE at different scan rates. The insets show the scan rate dependence of the peak current of the electrodes.
