# Supplementary material for: Enhanced Photovoltaic Performance of Poly(3,4-Ethylenedioxythiophene)Poly(N-Alkylcarbazole) Copolymer-Based Counter Electrode in Dye-Sensitized Solar Cells
Source: Polymers (Basel). 2024 Oct 20;16(20):2941. doi: 10.3390/polym16202941 (PMC11510948; doi:10.3390/polym16202941)
Supplement: Supplementary file 1 [file polymers-16-02941-s001.zip › polymers-3206242-supplementary.pdf]

Supplementary Materials

Enhanced photovoltaic performance of PEDOT - Poly(*N*-alkyl-carbazole) copolymer-based counter electrode in dye-sensitized solar cells

Sherif Dei Bukari, Aliya Yelshibay, Bakhytzhan Baptayev, and Mannix P. Balanay**

9H-carbazole

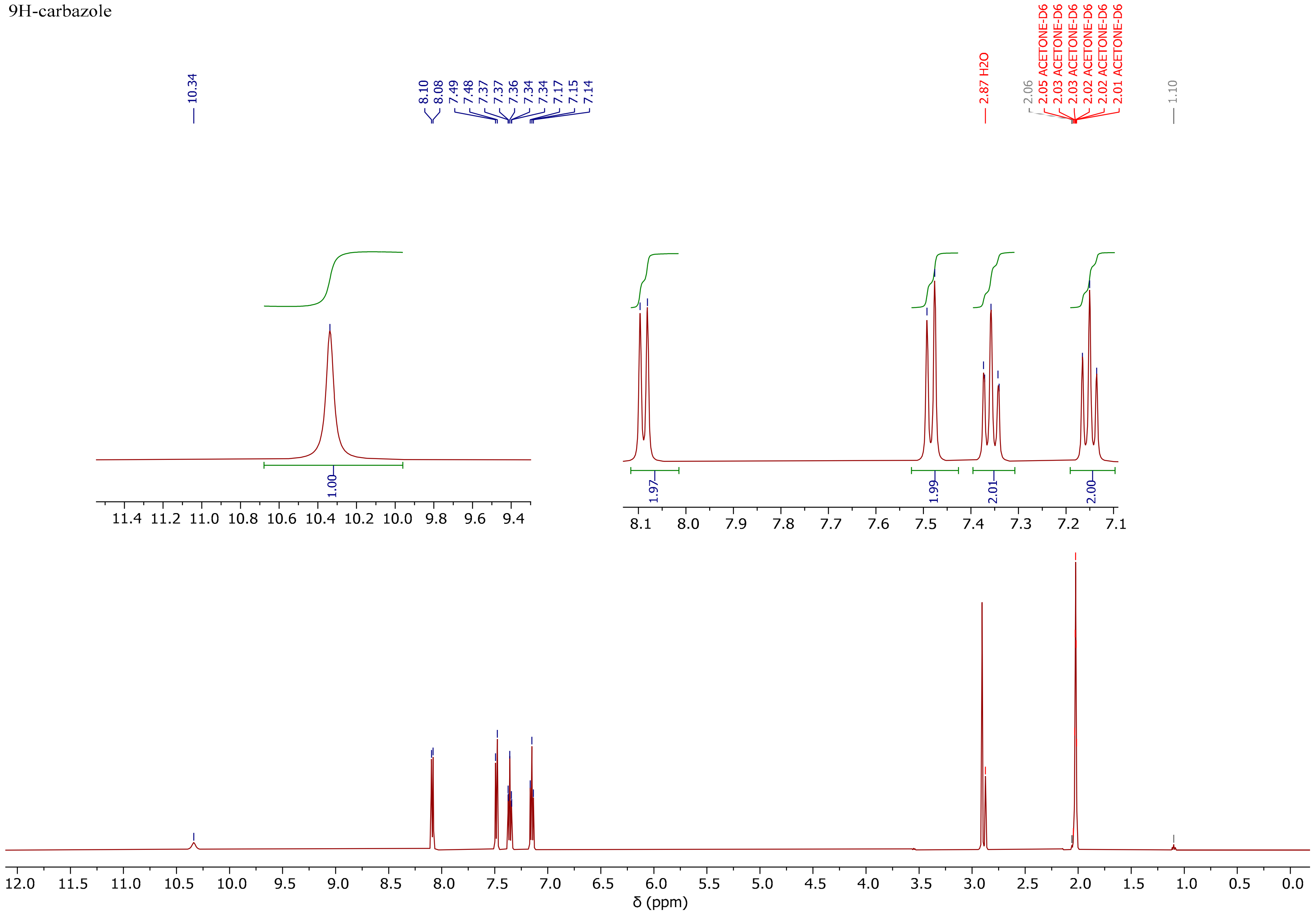

Figure S1. ^{1}H NMR spectrum of 9*H*-carbazole in acetone-d_6.

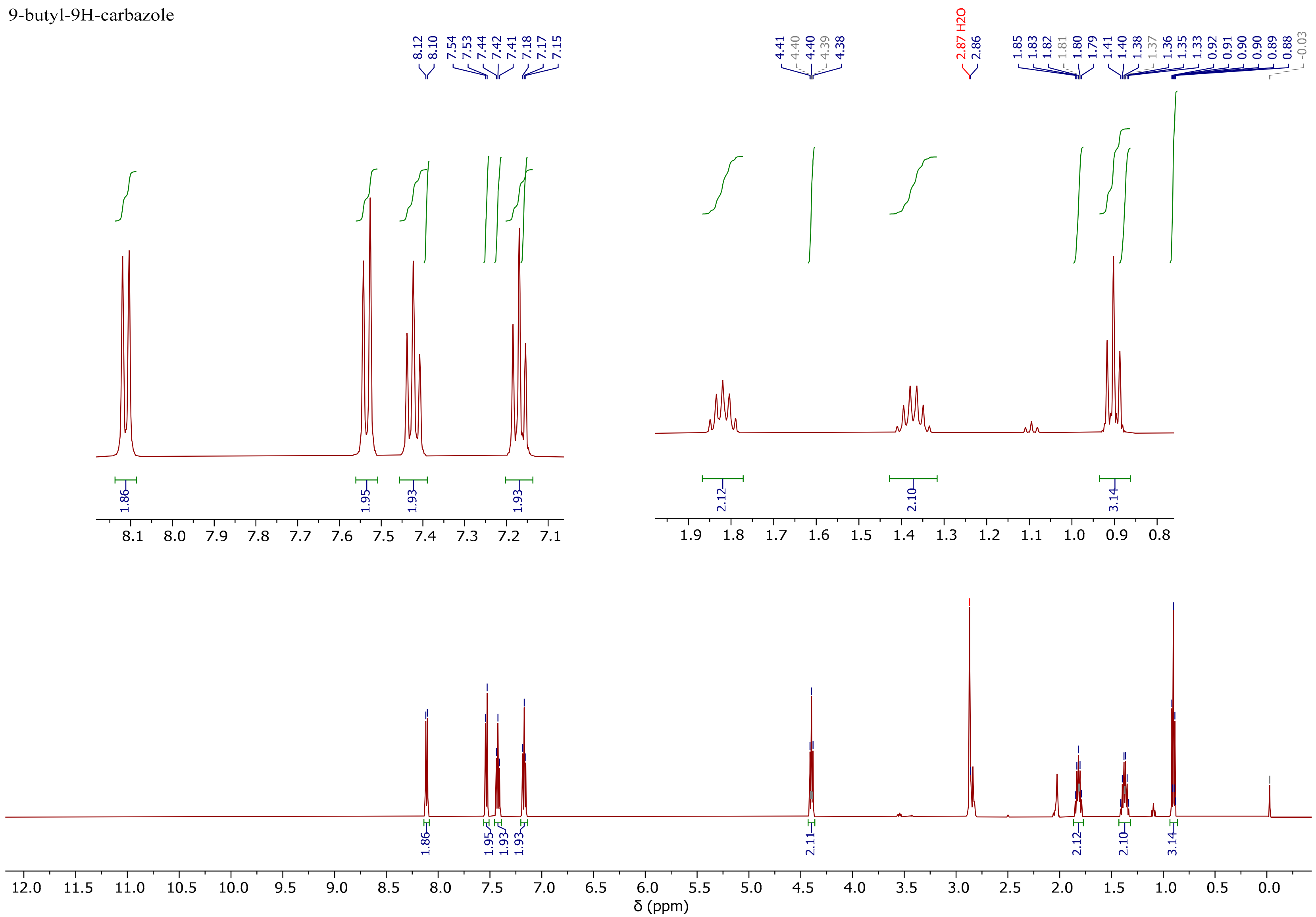

Figure S2. ^{1}H NMR spectrum of CbzC4 in acetone-*d*$_6$.

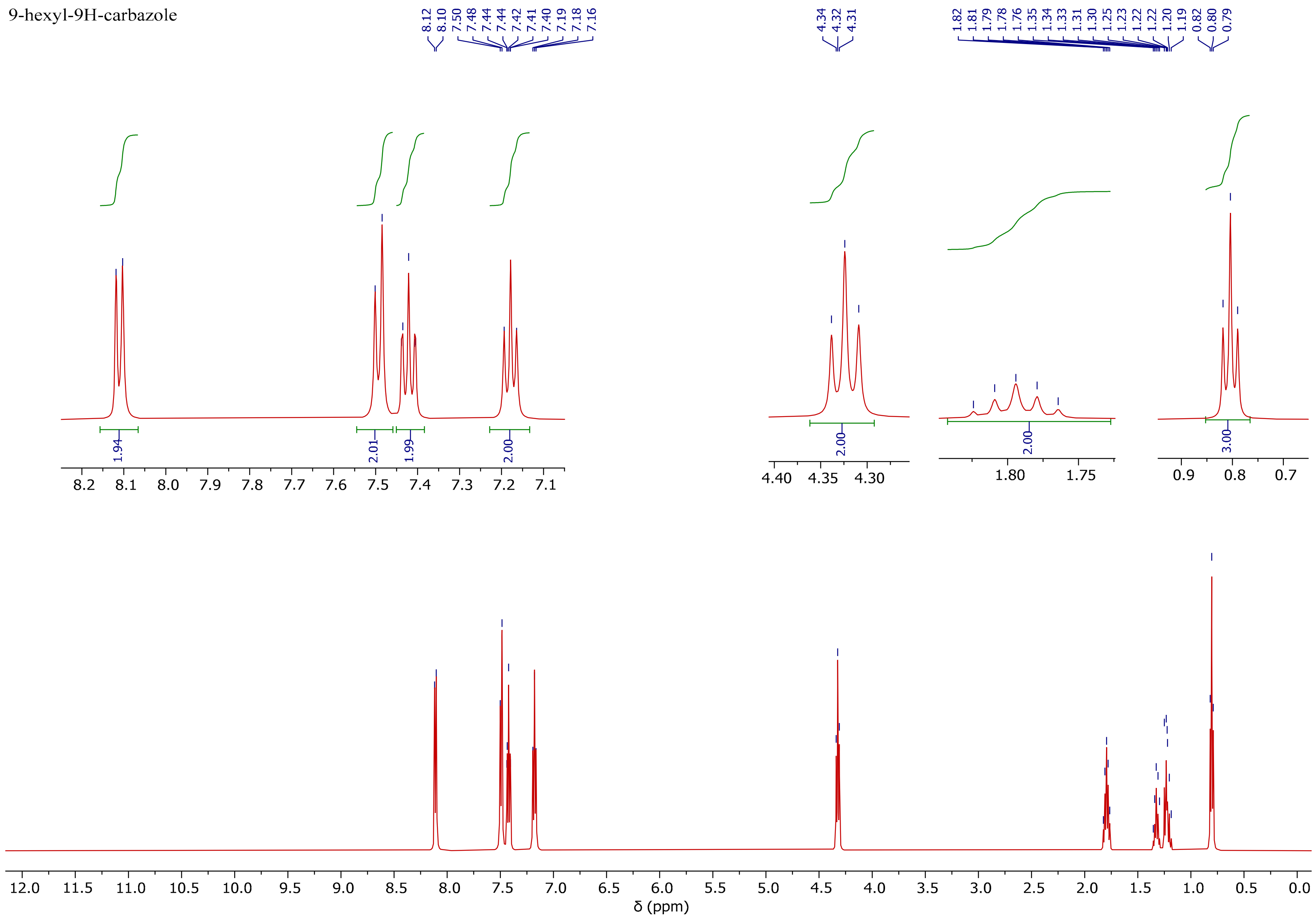

Figure S3. ^{1}H NMR spectrum of CbzC6 in acetone-d_6.

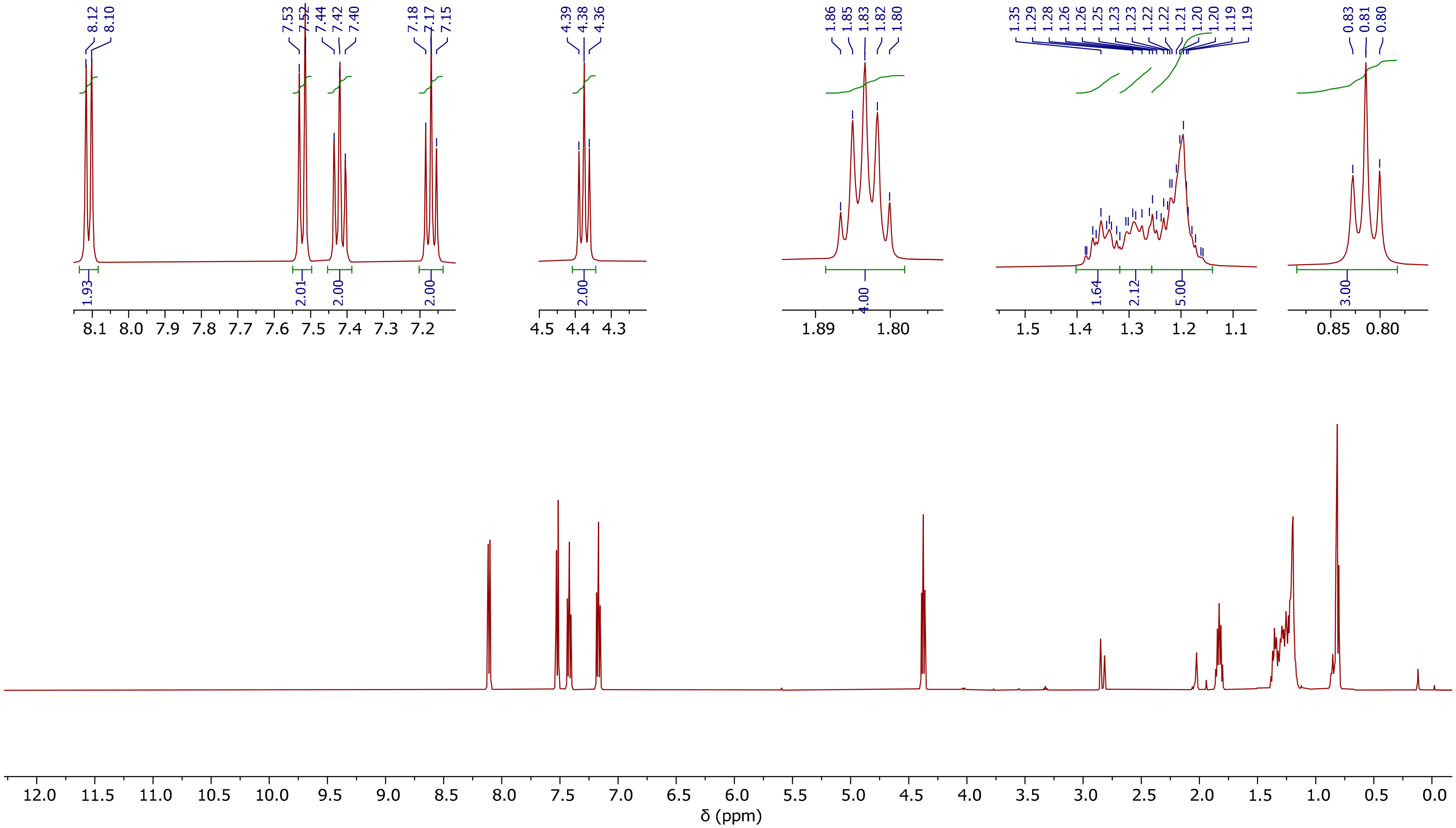

Figure S4. ^{1}H NMR spectrum of CbzC8 in acetone-d_6.

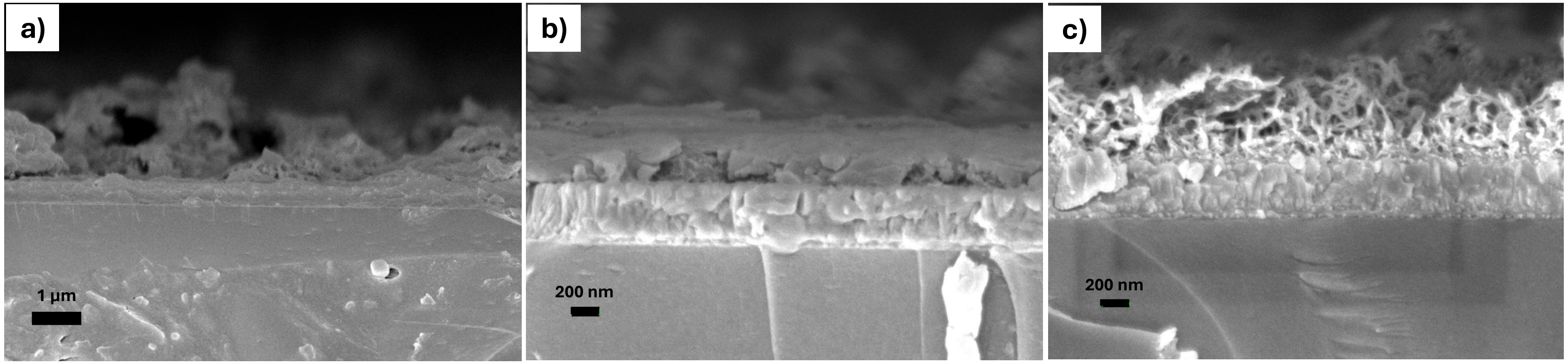

Figure S5. The SEM cross-sectional images of a) PEDOT, b) PCbzC8 and c) PEDOT-PCbzC8 thin films on FTO glass substrate

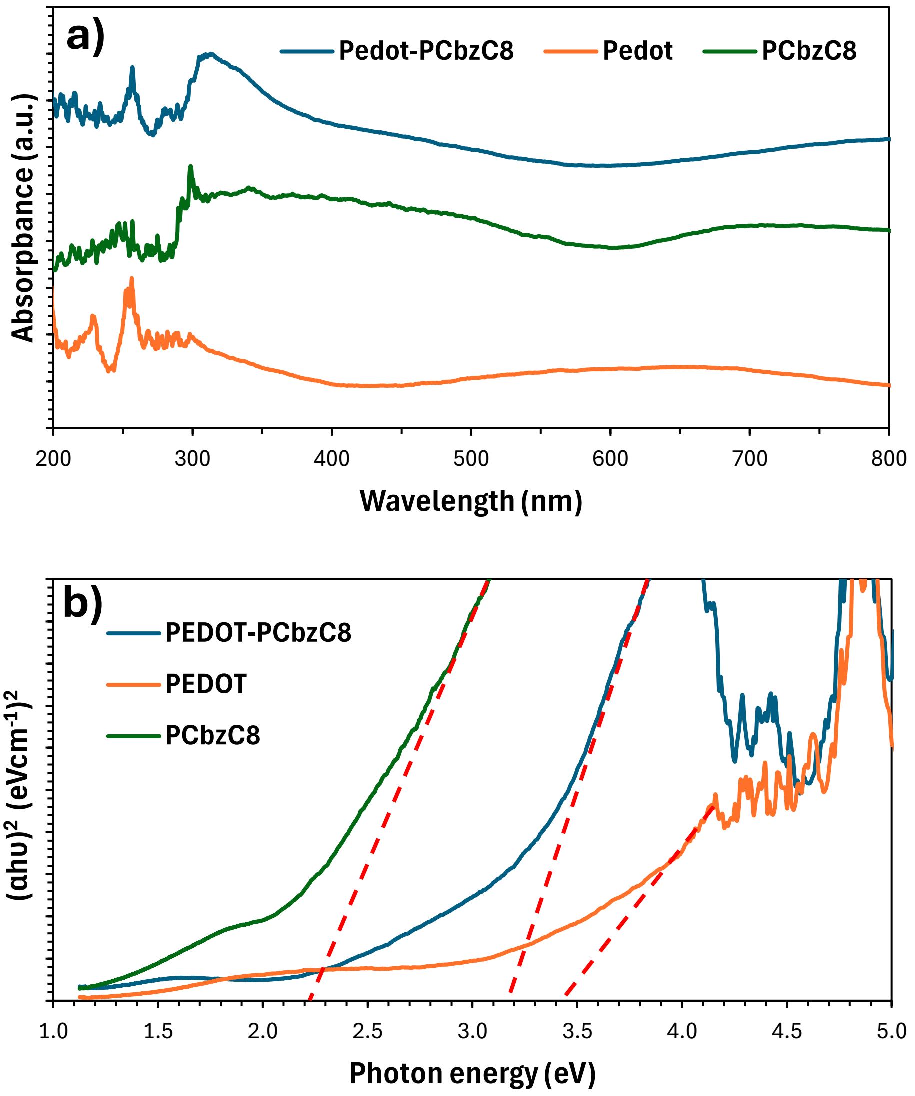

Figure S6. a) UV-Vis absorption and b) Tauc plot of polymer thin films on FTO glass substrate

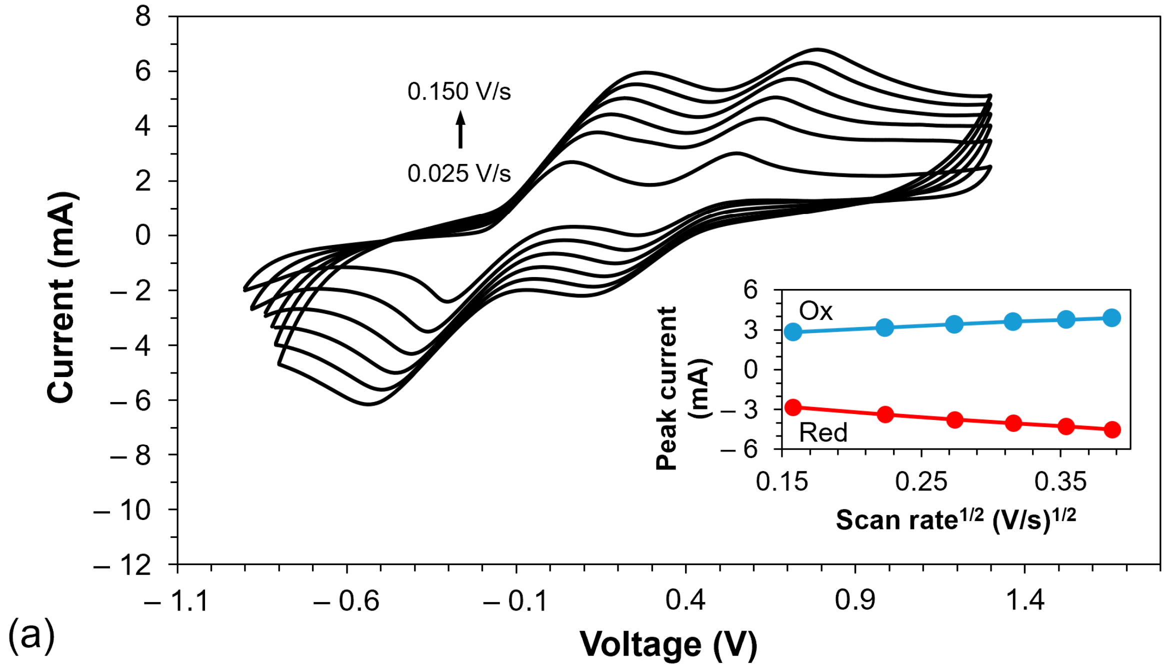

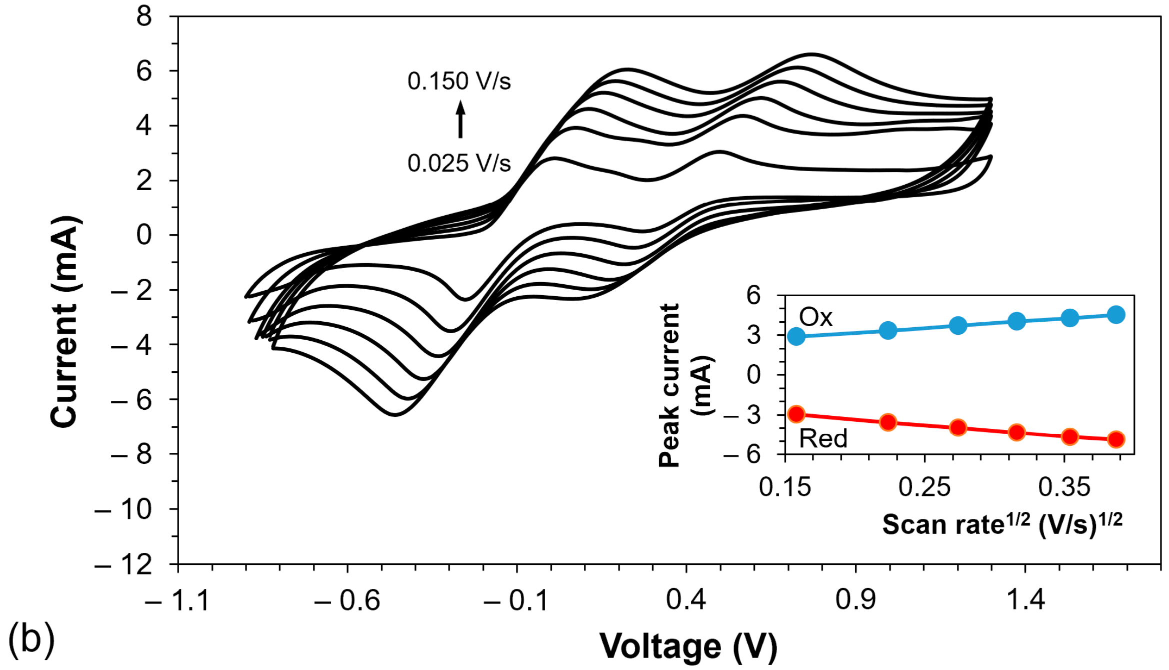

Figure S7. Cyclic voltammogram of **(a)** PEDOT-PCbzC4 and **(b)** PEDOT-PCbzC6 CE at different scan rates. The insets show the scan rate dependence of the peak current of the electrodes
